# Potential Apoptotic Activities of *Hylocereus undatus* Peel and Pulp Extracts in MCF-7 and Caco-2 Cancer Cell Lines

**DOI:** 10.3390/plants11172192

**Published:** 2022-08-24

**Authors:** Hanin S. Salam, Mohamed M. Tawfik, Mohamed R. Elnagar, Hamdoon A. Mohammed, Mohamed A. Zarka, Nabil S. Awad

**Affiliations:** 1 College of Biotechnology, Misr University for Science and Technology, Giza 12563, Egypt; 2 Zoology Department, Faculty of Science, Port Said University, Port Said 42526, Egypt; 3 Department of Pharmacology and Toxicology, Faculty of Pharmacy, Al-Azhar University, Cairo 11823, Egypt; 4 Department of Pharmacology, College of Pharmacy, The Islamic University, Najaf 54001, Iraq; 5 Department of Medicinal Chemistry and Pharmacognosy, College of Pharmacy, Qassim University, Buraydah 51452, Saudi Arabia; 6 Department of Pharmacognosy and Medicinal Plants, Faculty of Pharmacy, Al-Azhar University, Cairo 11823, Egypt; 7 Department of Pharmacognosy, Faculty of Pharmacy, October University for Modern Sciences and Arts (MSA), 6th October City, Giza 12563, Egypt; 8 Department of Pharmacognosy, College of Pharmacy, The Islamic University, Najaf 54001, Iraq; 9 Department of Genetics, Faculty of Agriculture and Natural Resources, Aswan University, Aswan 81528, Egypt

**Keywords:** *Hylocereus undatus*, phenolic acids, flavonoids, pitaya, MCF-7, Caco-2, apoptosis, p53, Bcl-2 mRNA, anticancer

## Abstract

There is a huge demand for novel anticancer agents with fewer side effects compared to current therapies. Pitaya, or dragon fruit, is a reservoir of potent anticancer compounds. This research aimed to analyze the phytochemical components of *Hylocereus undatus* pulp and peel extracts using LC-MS and GC-MS, and to investigate the in vitro effects of both extracts against cancer (breast, MCF-7, and colon, Caco-2) and normal (lung; WI-38 and breast; MCF-10A) cell proliferation using the MTT assay. The apoptosis potential of the anticancer effects was also evaluated using flow cytometry, RT-PCR, and Western blot. The total phenolic and flavonoid contents in the peel extract were significantly higher than those in the pulp extract. Compared to the flavonoid and phenolic acid standards, the LC-MS analysis revealed the presence of nine compounds, which were represented as 84.32 and 5.29 µg/g of the flavonoids and 686.11 and 148.72 µg/g of the phenolic acids in the peel and pulp extracts, respectively. Among the identified compounds, chlorogenic acid, caffeic acid, ferulic acid, and rutin were found at the highest concentration in both plant extracts. Both extracts displayed cytotoxic activity against MCF-7 and Caco-2 cancer cells after 48 h of treatment at IC_50_ values ranging from 14 to 53 μg/mL with high selective indices against normal WI-38 and MCF-10A cell lines. The increase in apoptosis was revealed by the overexpression of p53, BAX, and caspase-9 and the downregulation of antiapoptotic Bcl-2 mRNA and protein expressions. The results indicate that *H. undatus* extracts can be a plant source for cancer therapy.

## 1. Introduction

Breast and colorectal cancers are the most prevalent causes of malignancy and mortality worldwide. They caused about 1.6 million deaths and contributed 21.7% to the total number of newly diagnosed cancer cases in 2020 [1]. Notably, the incidence and mortality rates of breast and colorectal cancers in Arab people have risen over the past two decades [2,3]. Surgical operations, radiotherapy, chemotherapy, and hormone-based therapy are the mainstay treatments for these types of cancer [4,5]. However, several risk factors limit the use of these classical and routine cancer therapies, such as the ability of tumor cells to develop resistance and chemotherapy-induced toxicities [6,7]. Therefore, the discovery and development of novel anticancer agents have become a research necessity.

The global focus has been massively directed toward natural products as a rich source of anticancer agents with comparable efficacy to conventional treatment and relatively mild side effects [8,9]. Several FDA-approved natural product-based drugs are used in anticancer therapy, more than half of which are derived from plant sources [10,11].

Of about 300,000 angiosperms, approximately 80% have not been previously investigated for their medicinal activities [12,13]. One such plant family with promising medical value is the cactus family—Cactaceae. Cactaceae are adapted to dry environments, which may enhance their production of bioactive agents potentially valuable for drug discovery and development [14,15,16,17]. Cactus oil is rich in unsaturated essential fatty acids, such as palmitic acid and oleic acid, with potent antimicrobial, anti-inflammatory, cardioprotective, and antioxidant effects. To date, only a few cacti have been examined for their anticancer activities [18,19,20,21,22].

White pitaya (*Hylocereus undatus*) has been widely marketed as an edible fruit crop (dragon fruit) and is being cultivated across tropical regions of the world [23]. White pitaya fruit is high in antioxidants, such as phenolics, flavonoids, and vitamins, such as ascorbic acid [24]. Reported LC-MS analysis of the fruit’s pulp has indicted the presence of hydroxybenzoic and cinnamic acid derivatives, e.g., gallic acid, syringic acid, chlorogenic acid, caffeic acid, coumaric acid, ferulic acid, and dicaffeoylquinic acid [24]. Several flavonoids in the glycosidic and free aglycone forms, e.g., epicatechin, quercetin, rutin, typhaneoside, kaempferol, diosmin, baicalein, and isorhamnetin, were also identified in the fruit pulp extract [24]. The fruit pulp is also a rich natural source of betacyanins, e.g., hylocerenin, isohylocerenin, and phyllocactin, which are known for their broad biological activities, including antioxidant and antibacterial activities, and seem to contribute to the fluorescent color of the fruit pulp [25]. However, few studies have looked into white pitaya’s anticancer properties. Therefore, this research aimed to profile the phytochemical constituents of *H. undatus* pulp and peel extracts and their apoptosis-inducing activities against breast and colorectal cancer cell lines.

## 2. Results

### 2.1. The Phenolic and Flavonoid Contents of H. undatus Peel and Pulp Extracts

As equivalents to gallic acid (GAE) and quercetin (QE), the total phenolic content (TPC) and total flavonoid content (TFC) of *H. undatus* peel and pulp extracts were measured and are presented in Table 1. The results showed that the phenol concentration in the pulp extract was about seven times lower than that in the peel extract. Similarly, the TFC was higher in the peel extract (3.5 mg/g) than in the pulp extract (0.11 mg/g) (Table 1).

### 2.2. Gas Chromatography–Mass Spectrometry (GC-MS) Analysis of the Peel and Pulp Extracts of H. undatus

A total of twenty-two and twenty-three compounds were identified in the GC-MS chromatograms of peel and pulp extracts of the plant, respectively. The identification processes were primarily based on the retention time and mass fragments of the peaks compared to the Wiley spectral library collection and the NSIT library database. The relative percentages of the compounds were also calculated according to the individual peak areas of each compound compared to the total peak areas in the GC-chromatogram (Table 2). Interestingly, the most prevalent compound in the peel extract was 9-octadecenoic acid (Z), with a retention time of 26.95 min and a peak area of 13.22%. Propanoic acid anhydride was the most dominant compound in the peel extract, with a retention time of 5.66 min and a peak area of 25.36%. The peel and pulp extracts of *H. undatus* share only nine similar phytocompounds in convergent percentages. The most prevalent compounds are 9-octadecenoic acid (Z)—(peel: 13.22%, pulp: 8.85%)—and 1,3,5-trimethylbenzene (peel: 9.32%, pulp: 9.11%). The mass spectrometry of the peel extract revealed 13 compounds that were not detected in the pulp extract. The most dominant were 4-methylpentanoic acid (11.19%), 1-ethyl-4-methylbenzene (9.98%), and 1,3-propanediol, TBDMS derivative (8.9%). The GC analysis also indicated that fourteen compounds identified in the mass spectrum of the pulp extract were not found in the mass spectrum of the peel extract. The most dominant were propanoic acid, anhydride (25.63%), and 2-butoxyethanol (18.14%).

### 2.3. Liquid Chromatography Mass Spectrometry Analysis of the Extracts

The LC-MS analysis of the peel and pulp extracts of H. undatus was conducted using specific phenolic acid and flavonoid standards. As demonstrated in Table 3, eight and nine compounds were identified in the pulp and peel extracts of the plant, respectively. The identification of the compounds was carried out based on their retention time and mass spectral analysis compared to phenolic acid and flavonoid euthenics (the list of the phenolic acid and flavonoid standards used in the analysis is mentioned in the materials and methods, Section 4.2.4). The quantity of each identified compound was calculated based on its peak area compared to the peak area of the corresponding standard. The total quantity of the identified compounds in the peel extract was found to be 770.43 µg/g of the plant extract. The plant peel extract contained three flavonoids—rutin, quercetin, and kaempferol (a total of 84.32 µg/g)—and six phenolic acids: 3,4-dihydroxybenzoic acid, chlorogenic acid, caffeic acid, coumaric acid, vanillin, and ferulic acid (a total of 686.11 µg/g). The LC-MS analysis also showed that there is a similarity between peel and pulp extracts with regard to the identified compounds. However, proportions of these compounds were found to be noticeably different in both extracts of the plant. All the detected compounds in peel extract, except for quercetin, were also detected in the pulp extract of the plant. In addition, these compounds were measured at lower concentrations in the pulp extract compared to their concentrations in the peel extract of the plant (Table 3). The total concentration of the identified phenolic acids and flavonoids in the pulp extract was found to be 148.72 and 5.29 µg/g, respectively. The mass fragmentation pattern of the identified compounds was also used to confirm the compounds’ identities. For instance, rutin, which was found at a concentration of 72.45 and 5.24 µg/g in the peel and pulp extracts, respectively, was shown to have a mass parent ion *m*/*z* of 609 [M]- and fragments of 301 [M-H-rutenoside]- and 300 [M-2H-rutenoside]-, which further confirms the rutin structure identity [26]. Flavonoid aglycone fragmentation patterns were also found to be similar to the literature. For example, quercetin showed fragment peaks at *m*/*z* 151 and 178, the two main fragments reported for quercetin in the negative ion mode mass analysis [27].

The identified phenolic acids and flavonoids have been comprehensively reported for their cytotoxic and anticancer activities. Chlorogenic acid, which was identified in high concentrations in the plant, has been reported for its potential therapeutic usage in breast cancer [28]. In addition, other detected compounds, i.e., caffeic acid, coumaric acid, quercetin, kaempferol, and rutin, have been found in several plant extracts with potential antiproliferative activity [29,30].

### 2.4. Cell Proliferation by MTT Assay

**Figure 1:**
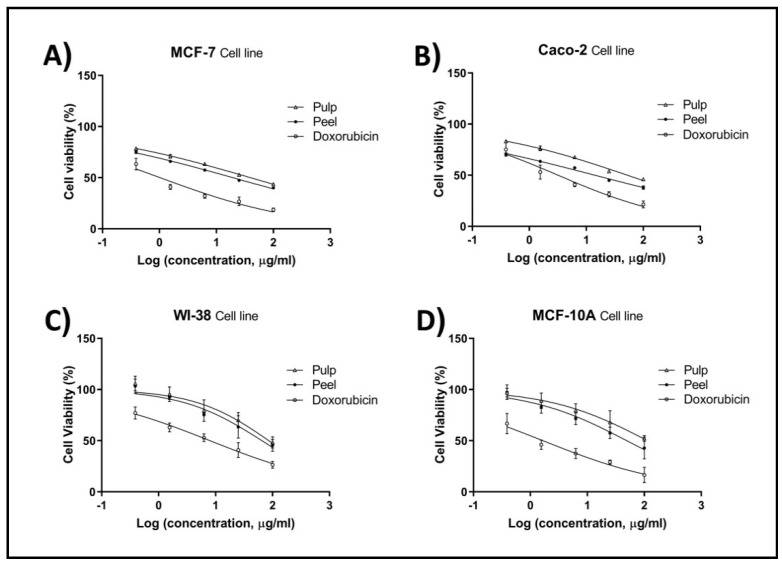
Effects of the pulp and peel extracts of *H. undatus* on the proliferation of MCF-7 (**A**), Caco-2 (**B**), WI-38 (**C**), and MCF-10A (**D**) cells. Untreated cells were used as control.

The proliferation of breast (MCF-7) and colorectal (Caco-2) cancer cells was significantly inhibited in a dose-dependent manner after being treated with pitaya pulp and peel extracts for 48 h (Figure 1). The peel extract exhibited more antiproliferative activity than the pulp extract against MCF-7 and Caco-2 cells, with IC_50_ values of 19.47 and 14.20 µg/mL, respectively. Moderate cytotoxicity was detected in MCF-7 (IC_50_: 39.84 µg/mL) and Caco-2 (IC_50_: 52.79 µg/mL) cells after treatment with pulp extract (Table 3). Results also showed that pulp and peel extracts had higher IC_50_ values on both MCF-10A (IC_50_: 113.80 and 49.1 μg/mL, respectively), and WI-38 (IC_50_: 86.01 and 65.16 μg/mL, respectively) normal cell lines. Although IC_50_ values of pulp and peel extracts were far higher than doxorubicin on both MCF-7 and Caco-2, both extracts were found mostly selective towards cancer cells (Table 4). This result drove us to further explore the potential antitumor effect of pulp and peel extracts.

### 2.5. Annexin-V/PI Double-Staining Assay

**Figure 2:**
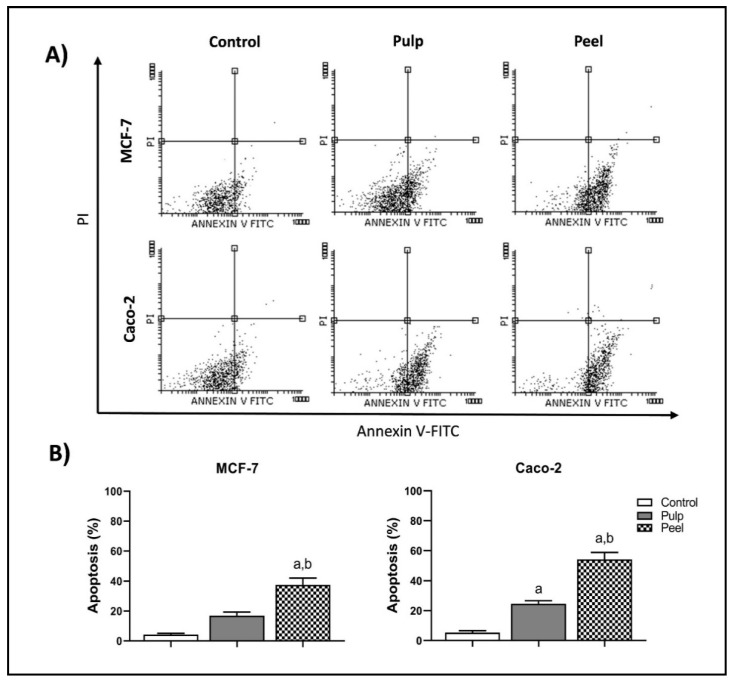
Flow cytometric analysis of apoptosis in MCF-7 and Caco-2 cells exposed to IC_50_ of pulp and peel extracts of *H. undatus* for 48 h, stained with annexin-V/propidium iodide (PI) and analyzed for apoptosis using Flowing Software. (**A**) Representative scatter plots of PI vs. annexin-V. The viable cells (An ^−^, PI ^−^) are shown in the lower left quadrants, while the early apoptotic cells (An ^+^, PI ^−^) are shown in the lower right quadrants. The upper left quadrants contain the necrotic cells (An ^−^, PI ^+^), while the upper right quadrants demonstrate the late apoptotic cells (An ^+^, PI ^+^). (**B**) Apoptosis is quantified as the mean ± SEM of three independent experiments. a: *p* < 0.05, significantly different from the corresponding control group. b: *p* < 0.05, significantly different from the pulp extract-treated group. Multiple comparisons were accomplished using one-way ANOVA followed by Tukey–Kramer post hoc analysis.

Both the pulp and peel extracts of *H. undatus* induced apoptotic cell death in MCF-7 and Caco-2 cells, with significant preference for the peel extract. After 48 h of treatment with the IC_50_ of both extracts, a highly significant increase in the apoptotic cell population was observed compared to the control cells (*p* < 0.05). In the MCF-7 cells, the percentage of early apoptotic cells significantly increased from 4.15% in untreated cells to 16.50% and 37.17% after being treated with the pulp and peel extracts, respectively (Table 5) (*p* < 0.05). Both extracts induced a statistically significant increase in the percentage of early apoptotic cells from 5.15% in the control cells (untreated cells) to 24.20% and 53.87% in the Caco-2 cells treated with the pulp and peel extracts, respectively (*p* < 0.05) (Figure 2).

### 2.6. Real-Time PCR Analysis

**Figure 3:**
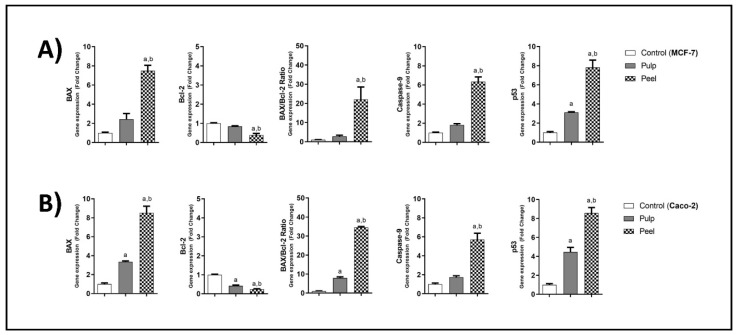
Gene-expression analysis of BAX, Bcl-2, caspase-9, and p53 after treatment with IC_50_ of *H. undatus* pulp and peel extracts for 48 h of (**A**) MCF-7 and (**B**) Caco-2 cells. Cells were seeded in T-25 cm^2^ tissue culture flasks for 24 h before treatment with DMSO or with pulp or peel extracts for 48 h. Total RNA was extracted, reverse-transcribed, and assayed for BAX, Bcl-2, Caspase-9, and p53 gene expression by quantitative reverse-transcription PCR. GAPDH was used as the housekeeping gene. Data from at least three independent experiments are presented as a ratio of the target gene/GAPDH expression (relative mRNA levels) and are represented as the mean ± SEM. Normalized data are expressed as fold changes, with the control set to “1.” a: Significantly different from the corresponding control group at *p* < 0.05. b: Significantly different from the pulp extract-treated group at *p* < 0.05. Multiple comparisons were accomplished using one-way ANOVA followed by Tukey–Kramer analysis as a post-ANOVA test.

Real-time PCR (RT-PCR) was conducted to assess the mRNA-expression levels of various apoptotic cell death-regulatory genes in MCF-7 and Caco-2 cancer cells following treatment with *H. undatus* pulp and peel extracts. The expression of all the proapoptotic genes tested, namely, BAX, caspase-9, and p53, was significantly increased by varying folds in Caco-2 and MCF-7 cells treated with peel and pulp extracts at *p* < 0.05. The peel extract induced the expression of the proapoptotic genes more than the pulp extract. A significant increase of about sevenfold and eightfold in BAX and p53 expression, respectively, was observed in both Caco-2 and MCF-7 cells treated with the peel extract at *p* < 0.05. The mRNA expression of the prosurvival gene Bcl-2 significantly decreased in the treated cells (Figure 3).

### 2.7. Western Blot Analysis

**Figure 4:**
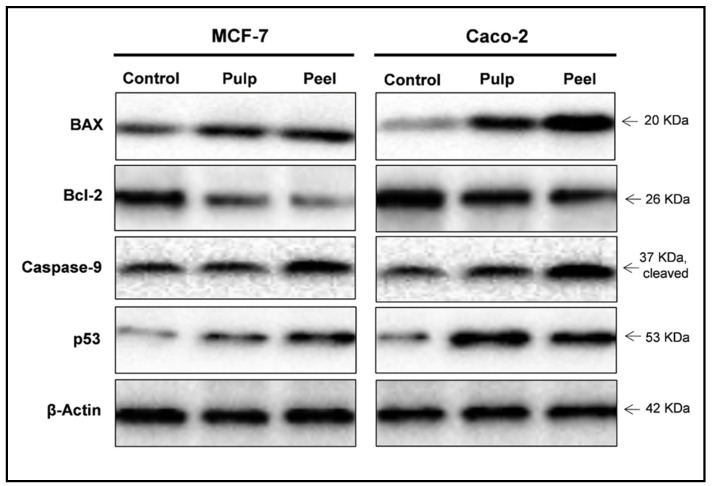
The immunoblotting images for the expression levels of BAX, Bcl-2, cleaved caspase-9, and p53 proteins, with β-actin as loading control. Representative Western blot images show the effects of the pitaya pulp and peel extracts on the expression levels of BAX, Bcl-2, caspase-9, and p53 proteins in MCF-7 and Caco-2 cells.

Next, Western blotting was performed to investigate the effect of the peel and pulp extracts on the expression levels of apoptosis-related proteins. The expression of the antiapoptotic Bcl-2 protein remarkably decreased in MCF-7 and Caco-2 cells 48 h after treatment with IC_50_ concentrations of pitaya peel and pulp extracts (Table 6). Expression of the apoptosis-inducing proteins BAX, caspase-9, and p53 increased in the treated cells. Upon treatment of MCF-7 and Caco-2 cells with both extracts, notable increases in Bax/Bcl-2 protein-expression ratios were observed compared with the untreated control cells (Figure 4).

## 3. Discussion

Herbal medicines have attracted the attention of health-care researchers globally to discover and develop new anticancer candidates. Medicinal plants are a promising source of potent, cheap, and readily available anticancer drugs with fewer side effects compared with classical chemotherapeutics [31,32,33]. A significant number of fatty acids in the oil of plants belonging to the Cactaceae family have potential anticancer activities [24,34,35]. A few studies have recently evaluated the antitumor efficacy of dragon fruit (*H. undatus*) extracts, but its cancer cell-death mechanism remains unknown [36,37].

LC-MS analysis has been conducted and revealed the presence of higher concentrations of phenolics and flavonoids in the peel extract when it was compared to the pulp extract of the plant (Table 3). The LC-MS analysis results are consistent with the spectroscopic analysis results for total phenolic and flavonoid contents. Chlorogenic and caffeic acids present as the dominant polyphenols found in studied extracts. Peel extract exhibits three to five folds higher yield of both acids than the yield of pulp extract. Chlorogenic and caffeic acids are very common phenolic compounds well known for their anticancer activities by inducing apoptosis [38,39]. Caffeic acid has been observed in previously studied *Hylocereus* species [40]. GC-MS analysis of the peel and pulp extracts of *H. undatus* allowed for the identification of their bioactive components. Among the identified compounds, the most common compound in the two extracts was the unsaturated fatty acid 9-octadecenoic acid (Z) (oleic acid). Oleic acid has been previously detected in the methanol extract of *H. undatus*, in agreement with our results [25]. This fatty acid has also been found in cactus fruits and seeds, such as in *Corryocactus brevistylus* [41] and *Opuntia ficus-indica* [35,42]. This fatty acid has been shown to have anticancer properties as well as the ability to induce apoptosis in cancer cell lines [43,44]. The findings of LC-MS and GC-MS may explain the ability of *H*. *undatus* peel and pulp extracts to inhibit the growth of MCF-7 and Caco-2 cancer cells, with some preference for the peel extract. Notably, the peel extract had much higher levels of fatty acids than the pulp extract. Likewise, the phytochemical analysis of *H*. *undatus* peel and pulp extracts revealed that the peel extract is richer in phenolic and flavonoid compounds than the pulp extract, which agrees with previous studies of dragon fruits [45,46]. It is well known that polyphenolic compounds, such as flavonoids, are one of the most important anticancer agents found in plants [47,48,49,50,51]. These secondary metabolites have been shown to induce apoptosis in cancer cells [52,53,54].

In the current study, lower cytotoxic activities of both extracts were detected against normal cell lines. The activity against the two tested cancer cell lines was up to sixfold greater at the IC_50_ concentration. Interestingly, *H. undatus* extracted by other solvents has been shown to have less antiproliferative effects against human hepatocellular, prostate, and gastric carcinoma cells [19,25,36].

Induction of apoptosis is one of the prospective strategies to enforce selective cell death in cancer cells, leading to valuable nonsurgical cancer treatment with fewer side effects [55,56]. The annexin-V and PI double-staining assay elucidated the efficacy of *H. undatus* peel and pulp extracts in triggering apoptosis in both Caco-2 and MCF-7 cells treated with IC_50_ concentrations, showing an increase in the percentage of early and late apoptotic cells compared to untreated cells. This is in agreement with previously reported experimental data for other pitaya and cactus species that can induce apoptosis in breast cancer and leukemia [54,57]. The high fatty acid and polyphenolic compound content of peel and pulp extracts may explain their apoptotic potential [42,44,51].

The RT-PCR and Western blot analysis in the present study elucidated the increased expression of proapoptotic molecules BAX, caspase-9, and p53 and the reduced expression of the antiapoptotic target Bcl-2 in both Caco-2 and MCF-7 cells treated with the IC_50_ of *H. undatus* peel and pulp extracts. Some of the identified fatty acids in both extracts, such as palmitic and oleic acids, exhibited high antiproliferation of breast and colon cancer cells through induction of apoptosis by caspase activation [53]. Several in silico studies have revealed the affinity of the interaction of palmitic and oleic acids and the target proteins (BAX, caspase-9 and Bcl-2) [58]. Bcl-2 is a family of antiapoptotic proteins, including Bcl-2 and BCL-X_L_, and proapoptotic proteins, including BAX and BAC. Bcl-2 protein is an important apoptotic regulator that promotes cell survival by either directly binding to BAX or preventing the activation of caspase proapoptotic proteins [59]. On the contrary, p53, a proapoptotic member, interacts with the antiapoptotic protein Bcl-2 and suppresses its inhibitory effects on BAX, promoting cell apoptosis through the release of cytochrome C [60]. Accordingly, it is suggested that the cell-death mechanism of breast and colorectal cancer cells treated with white pitaya is apoptosis-dependent, which may offer a promising therapeutic option for cancer treatment.

## 4. Materials and Methods

### 4.1. Collection of the Plant Samples and Extraction Procedure

White pitaya (dragon fruit) was purchased from the market. The fruit was identified by Dr. Nael Fawaz, the taxonomist at the Flora and Phytotaxonomy Research Department, Horticulture Research Institute, Agricultural Research Centre, Egypt. A voucher specimen of the plant was submitted and saved under the number 621 at the herbarium. Fresh peel was separated from the ripe fruit before cutting, and then the peel and pulp were cut into 2 mm pieces. Ethanol 95% was used in the extraction process using Soxhlet apparatus. The extract was concentrated under reduced pressure and stored in a −20 °C freezer [61]. All the chemical reagents used were molecular biology grade.

### 4.2. Phytochemical Analysis

#### 4.2.1. Total Phenolic Content (TPC)

The TPC of the plant peel and pulp extracts was carried out using a reported method [62]. A reaction mixture of extracts (1.6 mL of 0.1 mg/mL), sodium carbonate solution (10 %, 200 µL), and Folin–Ciocalteu reagent (diluted 1:5 with distilled water, 200 µL) was thoroughly mixed and measured at 760 nm after 30 min of incubation at RT in a dark place. A standard calibration curve of gallic acid was plotted and used in the calculation of the TPC in the plant peel and pulp extracts as mg GAE/g of the extracts.

#### 4.2.2. Estimation of Total Flavonoid Content

The TFC estimation of dragon fruit pulp and peel extracts was carried out using reported methods [63]. A reaction mixture of extracts in methanol (2 mL of 0.1 mg/mL), aluminum chloride solution (10%, 100 µL), and potassium acetate solution (0.1 mM in distilled water, 100 µL) was thoroughly mixed and measured at 415 nm after 30 min of incubation at RT in a dark place. A standard calibration curve of quercetin was plotted and used in the calculation of the TFC in the plant peel and pulp extracts as mg QE/g of the extracts.

#### 4.2.3. Gas Chromatography–Mass Spectrometry (GC-MS) Analysis

A TRACE GC Ultra Gas Chromatograph (Thermal Scientific, Waltham, MA, USA) with a thermo-mass spectrometer detector (ISQ Single Quadrupole Mass Spectrometer) was used for the GC-MS study. A TR-5 MS column (30 m, 0.32 mm i.d., and 0.25 m thick film) was used in the GC-MS system. The following temperature program was used: kept at 60 °C for 1 min, then increasing at 4.0 °C/min to 240 °C/min, held for 1 min, utilizing helium as the carrier gas at a flow rate of 1.0 mL/min and a split ratio of 1:10. At 210 °C, the injector and detector were maintained. The mixes (1 µL) were injected with diluted samples in hexane (1:10). Electron ionization (EI) at 70 eV was used to acquire mass spectra with an *m*/*z* range of 40–450. The plant constituents were identified using AMDIS software (www.amdis.net) and mass spectral matching to genuine standards (where available), the Wiley spectral library collection, and the NSIT library database.

#### 4.2.4. Liquid Chromatography–Mass Spectrometry Analysis of the Extracts

The LC-MS analysis of the peel and pulp extracts was performed using liquid chromatography–electrospray ionization–tandem mass spectrometry (LC-ESI-MS/MS) with an ExionLC AC system for separation of the extracts’ components. The detection of the components was performed on a SCIEX Triple QuadTM 5500+ MS/MS system equipped with electrospray ionization (ESI). The separation was performed using a Zorbax^®^ Eclipse Plus C-18 column (4.6 × 100 mm, 1.8 µm). The mobile phases consisted of two eluents—A: 0.1% formic acid in water; B: acetonitrile (LC grade). The mobile phase was programmed as: 2% B 0–1 min, 2–60% B 1–21 min, 60% B 21–25 min, and 2% B 25.01–28 min. The flow rate was 0.8 mL/min and the injection volume was 3 µL. For MRM analysis of the selected polyphenols, positive and negative ionization modes were applied in the same run with the following parameters: curtain gas 25 psi; IonSpray voltage 4500 and −4500 for positive and negative modes, respectively; source temperature 400 °C; ion source gas 1 & 2 were 55 psi with declustering potential—50; collision energy 25; and collision energy spread 10.

The compounds were identified based on their RT and mass fragmentations compared to standard phenolic acids (chlorogenic acid, gallic acid, caffeic acid, coumaric acid, vanillin, ellagic acid, 3,4-dihydroxybenzoic acid, cinnamic acid, methyl gallate, ferulic acid, and syringic acid), and flavonoids (rutin, naringenin, quercetin, hesperetin, myricetin, kaempferol, apigenin, catechin, and luteolin).

### 4.3. Cell Lines

In the present work, human Caco-2 (colon cancer), MCF-7 (breast cancer), normal WI-38 (human lung fibroblasts), and normal MCF10A (nontumorigenic human breast epithelial) cell lines were utilized. Cell lines were purchased from the Holding Company for Biological Products and Vaccines (VACSERA, Giza, Egypt). Caco-2 and MCF-7 cells were cultured in RPMI-1640 medium, while the normal fibroblasts WI-38 cells were cultured in high-glucose DMEM. Both cell culture media enhanced with 10% fetal bovine serum (FBS) and 1% penicillin–streptomycin mixture (100 IU/mL penicillin and 0.1 mg/mL streptomycin) were used. The culture media for the normal MCF10A cells were freshly prepared from DMEM/Ham’s F12 (1:1) immediately supplemented with epidermal growth factor (EGF; 0.02 µg/mL], hydrocortisone [0.5 µg/mL], cholera toxin [0.1 µg/mL], horse serum [5%], and penicillin–streptomycin mixture [1%]). For both MCF-7 and MCF10A, the individual culture medium was supplemented with 0.01mg/mL bovine insulin (Sigma). All cell culture steps were carried out in sterile conditions, and all the cells were incubated in a CO_2_ incubator with 5% CO_2_.

### 4.4. Antiproliferation Assay

The extracts were tested for antiproliferation and cytotoxicity against cancer and normal cell lines using 3-(4,5-dimethylthiazol-2-yl)-2,5-diphenyltetrazolium bromide (MTT) (Sigma, Burlington, MA, USA). All the extracts were dissolved in DMSO and were subsequently diluted in the culture medium before treatment of the cultured cells. Once the MCF-7, Caco-2, WI-38, and MCF-10A cells were 80–90% confluent, they were harvested by treatment with a solution containing 0.25% trypsin, thoroughly washed, and suspended in supplemented growth medium. Cells were plated in 200 μL of medium/well (2 × 10^4^/well) in 96-well plates. After incubation overnight, the cells were treated with different concentrations (100, 25, 6.25, 1.56, 0.39, and 0 µg/mL) of the extracts in RPMI-1640 for 48 h. In parallel, the cells were treated with 0.1% DMSO as a negative control. After 48 h, 100 μL of MTT was added, and the cells were incubated for 4 h. MTT-formazan was formed by metabolically viable cells, which dissolved in 150 μL of DMSO after 20 min. The absorbance was measured at 492 nm using a microplate reader, which is directly proportional to the number of living cells in culture [64]. Percentage cytotoxicity was calculated using the following formula:

Percentage inhibitory ratio (%) = [(control abs − blank abs) − (test abs − blank abs)]/[control abs − blank abs] × 100 (1)

The selectivity index, which indicates the cytotoxic selectivity (i.e., drug safety) for studied extracts against cancer cells versus normal cells was calculated from the following formula:

Selectivity index = IC_50_ calculated for normal cell/IC_50_ calculated for cancer cell. The SI values more than 2 were considered as high selectivity [65,66].

### 4.5. Annexin-V/Propidium Iodide Flow Cytometric Analysis

The Caco-2 and MCF-7 cells were cultured in 25 cm^2^ flasks overnight, divided into groups, treated with IC_50_ concentrations of extracts, and incubated for 48 h. The cells were then harvested by trypsinization, washed with ice-cold PBS, and stained using the FITC annexin-V/propidium iodide (PI) An Apoptosis Detection kit was used (BD Biosciences, San Diego, CA, USA), according to the manufacturer’s instructions. Flow cytometric analysis was performed using the Epics XL-MCL™ flow cytometer, and the data were analyzed using Flowing software (Turku Centre for Biotechnology, Turku, Finland).

### 4.6. Quantitative Real-Time PCR

All procedures were based on Kumar et al. (2017) with some modifications as follows: total RNA was extracted from both untreated and treated Caco-2 and MCF-7 cells using Trizol reagent, according to the manufacturer’s instructions. In brief, 1 μg of RNA was reverse-transcribed into cDNA. The cDNA obtained was amplified to examine the gene expression of BAX, Bcl-2, caspase-9, and p53. An internal control, GAPDH, was used as a standard for the RT-PCR reaction. The sequences of the primers used were as follows:

1. Bax F: 5′-CCCGAGAGGTCTTTTTCCGAG-3′
  Bax R: 5′-CCAGCCCATGATGGTTCTGAT-3′
2. Bcl-2 F 5′-CCTGTG GAT GAC TGA GTA CC-3′
  Bcl-2 R 5′-GAGACA GCC AGG AGA AAT CA-3′
3. Caspase-9 F: 5′-CTGAGCCAGATGCTGTCCCAT-3’
  Caspase-9 R: 5′-CCAAGGTCTCGATGTACCAGGAA-3′
4. p53 F 5′-CCCCTCCTGGCCCCTGTCATCTTC-3′
  p53 R 5′-GCAGCGCCTCACAACCTCCGTCAT-3′
5. GAPDH F 5′-GCA AGT TCA ACG GCA CGA TCA AG-3′
  GAPDH R 5′-CTA CTC AGC ACC AGC ATC ACC-3′

The PCR conditions were initial denaturation for 5 min at 94 °C, followed by 40 cycles of denaturation at 94 °C for 30 s; annealing at 51 °C (p53), 54 °C (Bcl-2), 63.5 °C (caspase-9), 61.5 °C (BAX), and 57 °C (GAPDH) for 40 s; and extension at 72 °C for 45 s, followed by final extension at 72 °C for 5 min. The quantitative RT-PCR was performed with a real-time PCR kit (Bioneer, Seoul, South Korea) using SYBR Green in a Mini Option thermocycler. A negative control of DEPC water was used instead of the cDNA template. The SYBR Green RT-PCR assay performed was according to Muller et al. (2002). The mRNA expression level was presented in relation to the expression of GAPDH. The mRNA levels were all normalized with GAPDH expression level. To evaluate the quality of the RT-PCR products, the melt curve was analyzed after each assay. The expression is relative to the measure using the ΔΔCT technique with GAPDH as the reference gene [54].

### 4.7. Western Blot Analysis

The Caco-2 and MCF-7 cells were cultured in 25-cm^2^ flasks overnight, divided into groups, treated with IC_50_ concentrations of extracts, and incubated for 48 h. The experiment was terminated by lysing the cells in cold lysis buffer (pH 7.4; 100 mM NaCl, 10 mM Tris, 25 mM EDTA, 25 mM EGTA, 1% (*v*/*v*) Triton X-100, and NP-40, with 1:300 protease inhibitor) (Sigma, Burlington, MA, USA). and phosphatase inhibitor (Roche, Basel, Switzerland) cocktails] [67,68]. The cells were then collected and sonicated for 2 × 15 s under cooling conditions. Before proceeding to Western blotting, the total protein content was measured using the Bradford technique. Equal amounts (25 g) of protein samples were boiled in SDS-loading buffer for 5 min, cooled on ice for 10 min, loaded into SDS–polyacrylamide gel electrophoresis (Cleaver, UK), and transferred onto PVDF membranes (Biorad, Hercules, CA, USA) for 35 min using a Semi-Dry Transfer Cell (Biorad, Hercules, CA, USA). To decrease non-specific protein interactions between the membrane and the antibodies, the membrane was blocked with 5% nonfat dry milk in TBS-T for 1 h at 27 °C. The membrane was incubated overnight at 4 °C with each primary antibody at the indicated dilution. Primary antibodies against BAX, Bcl-2, caspase-9 (1:1000, Cell Signaling Technology, Danvers, MA, USA), p53 (1:1000, Abcam), and β-actin (1:2000, Sigma-Aldrich) were used. The blots were washed three times for 10 min each with TBS-T. The membrane was incubated with the corresponding horseradish peroxidase-linked secondary antibodies (Dako) for another 1 h at room temperature, followed by washing three times for 10 min each with TBS-T. A Western ECL chemiluminescent substrate (Perkin Elmer, MA, USA) was applied to the blot according to the manufacturer’s recommendation, and the chemiluminescent signals were captured using the Chemi-Doc imager (Biorad, Hercules, CA, USA). The band intensities were measured and normalized to β-actin [69].

### 4.8. Data Analysis

Statistical analyses were performed using GraphPad Prism 8 analysis (GraphPad Software, Inc., San Diego, CA, USA). Significance differences between groups were detected using one-way ANOVA at *p* < 0.05.

## 5. Conclusions

Our findings highlighted the selective cytotoxic activities of pitaya peel and pulp extracts against breast and colorectal cancer cells with high selectivity compared with normal WI-38 and MCF-10A cell lines. Phytochemical analysis revealed the presence of phenolics and flavonoids, which were more concentrated in the peel extract than the pulp extract of the plant. The results also clearly showed that both extracts can induce apoptosis in vitro. These data calls for further research on the use of white pitaya for the treatment of breast and colorectal cancer. Future experiments may be conducted to investigate the anticancer activities of pitaya extracts on animal models and to recognize the active components that contribute to their anticancer activity.

## Figures and Tables

**Table 1:** Quantitative measurement of phenolics and flavonoids in the peel and pulp extracts of *H. undatus*.

	TPC (mg/g GAE)	TFC (mg/g QE)
Peel extract	22.8	3.5
Pulp extract	3.0	0.11

TPC stands for total phenolic content, which is equivalent to gallic acid in mg/g of plant extract; TFC stands for total flavonoid content, which is equivalent to quercetin in mg/g of plant extract.

**Table 2:** Phytocomponents identified by the GC-MS analysis in the peel and pulp extracts of *H. undatus*.

Peak No.	Retention Time (Minutes)	Compound Name	Peak Area (%)	
			Peel	Pulp
	4.02	1-propanol, 2-methyl-	1.05	NA
	4.19	Ribitol	2.26	NA
	4.44	2-methyl malonic acid	3.34	NA
	4.54	1,3-Propanediol, TBDMS derivative	8.90	NA
	4.57	Propanoic acid, 2-methylpropyl ester	NA	3.93
	4.66	l-Felinine	NA	4.08
	5.01	Pentanoic acid, 4-methyl-	11.19	NA
	5.07	Ethanol, 2-butoxy-	NA	18.14
	5.49	2,5-Methylene-d,l-rhamnitol	2.30	0.82
	5.59	Benzene, 1-ethyl-4-methyl-	9.98	NA
	5.66	Propanoic acid, anhydride	NA	25.63
	5.7	Benzene, 1,3,5-trimethyl-	3.23	4.08
	5.85	1,1-Cyclobutane dicarboxamide, 2-phenyl-N,N′-bis(1-phenylethyl)	1.89	NA
	5.94	2,2-Dimethyl-3-phenylpropanoic acid	NA	1.57
	6.1	1,3,5-trimethylbenzene	9.32	9.11
	6.57	Butanoic acid, 2-amino-4-(methylsulfinyl)-, (ñ)	3.27	NA
	6.62	Benzene, 1,3,5-trimethyl-	NA	1.76
	6.69	Citronellal	NA	1.80
	6.97	Acetic acid, Octyl ester	1.33	NA
	7.03	Tetradecane, 1-chloro-	NA	1.17
	7.18	10,12-Octadecadiynoic acid	NA	2.35
	7.76	Acetic acid, Octyl ester	1.67	NA
	7.8	1-tetradecanol	NA	1.63
	8.25	Nonanoic acid	NA	0.66
	9.09	9-octadecenoic acid (z)-	3.61	3.61
	9.11	Nonanoic acid	NA	2.81
	9.81	2-Myristynoyl pantetheine	NA	0.71
	15.13	i-Propyl 9,12,15-octadecatrienoate	1.27	1.62
	19.68	Retinal	3.32	NA
	20.80	Alanine, 3-(benzyloxy)-, l-	2.13	NA
	23.75	Oleic acid	NA	2.92
	24.2	9-octadecenoic acid (z)-	4.93	1.91
	26.95	9-octadecenoic acid (z)-	13.22	8.85
	27.34	Oleic acid	1.90	1.10
	30.05	9-Octadecenoic acid (Z)-, 2-hydroxy-1-(hydroxymethyl) ethyl ester	4.69	3.34
	33.27	4H-1-benzopyran-4-one, 2-(3,4-dihydroxyphenyl)-6,8di-á-d-glucopyranosyl-5,7dihydroxy	1.73	NA

**Table 3:** LC-MS analysis of the peel and pulp extracts of *H. undatus*.

Compounds	RT	*m*/*z* Parent Ion [M-H]-	*m*/*z* Daughter Ions	Peel Extract µg/g	Pulp Extract µg/g
3.4-Dihydroxybenzoic acid	5.72	153	109, 91	1.30	0.55
Chlorogenic acid	7.32	355	163, 145, 135, 117, 89	491.03	91.80
Caffeic acid	8.02	179	161, 135, 105	108.94	31.78
Coumaric acid	9.48	163	119, 93, 71	14.31	3.63
Vanillin	9.48	151	136, 123, 107	10.66	3.71
Rutin	9.69	609	301, 300, 271, 255, 179, 151	72.45	5.24
Ferulic acid	10.18	192.8	178, 149, 134, 117, 89	59.86	17.26
Quercetin	13.51	301	273, 245, 179, 151, 121, 107	11.09	ND
Kaempferol	15.29	284.7	257, 255, 239, 227, 211, 185, 117, 93	0.78	0.05

All compounds were identified by comparison with specific phenolic acid and flavonoid standards.

**Table 4:** IC_50_ values and selectivity index (SI) of peel and pulp *H. undatus* extracts against MCF-7, Caco-2, WI-38, and MCF-10A cell lines.

	* IC_50_ (µg/mL)				Corresponding SI			
	MCF-7	Caco2	WI-38	MCF-10A	WI-38/MCF-7	WI-38/Caco-2	MCF-10A/MCF-7	MCF-10A/Caco-2
Pulp	39.84	52.79	86.01	113.80	2.15	1.62	2.86	2.15
Peel	19.47	14.20	65.16	49.17	3.34	4.58	2.52	3.46
Doxorubicin	1.03	3.20	7.97	1.66	7.73	2.49	1.61	0.52

* IC_50_ values were determined by nonlinear fit of dose–response curve in GraphPad prism 8. The classic chemotherapeutic agent doxorubicin was used as cytotoxic reference drug in MTT assay.

**Table 5:** Effects of the pulp and peel extracts of *H. undatus* on different stages of the cell-death process of MCF-7 and Caco-2 cells after 48 h of treatment.

	Viable ^a^	Apoptosis ^a^		Necrosis ^a^
		Early	Late	
MCF-7	95.67 ± 0.81	4.15 ± 0.42	0.13 ± 0.02	0.11 ± 0.03
Pulp	82.85 ± 0.90	16.50 ± 2.32	0.42 ± 0.07	0.20 ± 0.02
Peel	62.25 ± 4.53	37.17 ± 3.49 ^a,b^	0.38 ± 0.09	0.17 ± 0.01
Caco-2	94.60 ± 1.31	5.15 ± 1.33	0.13 ± 0.01	0.11 ± 0.02
Pulp	75.18 ± 2.05	24.20 ± 2.01 ^a^	0.40 ± 0.08	0.18 ± 0.01
Peel	45.61 ± 4.66	53.87 ± 4.68 ^a,b^	0.30 ± 0.07	0.18 ± 0.02

Values are given as the mean ± SEM of three independent experiments. ^a^ Significantly different from the corresponding control group at *p* < 0.05, ^b^ Significantly different from the pulp extract-treated group at *p* < 0.05.

**Table 6:** Effect of pitaya peel and pulp extracts on BAX, Bcl-2, caspase-9, and p53 protein expression levels in MCF-7 and Caco-2 cells 48 h after treatment.

	Protein Expression (Normalized to β-Actin) ^a^				
	BAX	Bcl-2	BAX/Bcl-2 Ratio	Caspases-9	p53
MCF-7	1.00	1.00	1.00	1.00	1.00
Pulp extract	1.49	0.66	2.22	1.25	1.44
Peel extract	2.64	0.39	6.14	2.22	2.63
Caco-2	1.00	1.00	1.00	1.00	1.00
Pulp extract	2.20	0.70	3.14	1.26	2.25
Peel extract	3.70	0.62	5.96	2.30	2.01

**^a^** Values are presented as changes from the corresponding untreated cells, which was set to “1.”

## Data Availability

The data presented in this study are available on request from the corresponding author.
